# Tumor-related gene expression levels in thymic carcinoma and Type B3 thymoma

**DOI:** 10.1186/s13019-016-0468-1

**Published:** 2016-05-26

**Authors:** Yoko Karube, Satoru Kobayashi, Sumiko Maeda, Tetsu Sado, Hiromi Ishihama, Masayuki Chida

**Affiliations:** Deparment of General Thoracic Surgery, Dokkyo Medical University, 880 Kitakobayashi, Mibu, 321-0294 Japan; Tumor Center, Dokkyo Medical University Hospital, Mibu, 321-0293 Japan

**Keywords:** Thymic carcinoma, Thymoma, Type B3 thymoma, Chemosensitivity

## Abstract

**Background:**

Thymic carcinoma (TC) is a rare type of malignant neoplasm that develops in the anterior mediastinum and associated with poor prognosis. Type B3 thymoma (B3) occasionally demonstrates malignant tumor characteristics, especially in the advanced stage. We investigated the expressions of tumor-related genes in resected TC and B3 specimens.

**Methods:**

TC and B3 specimens resected from 1999 through 2012 were investigated. Tumor segments were collected from the specimens by micro-dissection to extract mRNA, then RT-PCR was performed according to Dannenberg’s tumor profile method for semi-quantitation of tumor-related gene mRNA. To compare with other types of cancer, data from lung cancer (LC) cases in our database were also examined.

**Results:**

The gene expression levels of thymidylate synthase were significantly higher in TC and B3 as compared to LC specimens (*p* < 0.02), while no difference were observed between TC and B3 tumors. The ratio of folypolyglutamyl synthase (FPGS) to gamma-glutamyl hydrolase (GGH) mRNA was significantly lower in TC than in B3 (*p* < 0.05), with lower FPGS/GGH in those tumors related to overall survival. Also, the gene expression of vascular endothelial growth factor (VEGF) was significantly higher in TC as compared to B3 (*p* = 0.04), with higher VEGF gene expression in TC and B3 specimens related to overall survival of affected patients. Epidermal growth factor receptor (EGFR) expression was significantly higher in B3 as compared to both TC and LC specimens (*p* < 0.01). However, there were no EGFR gene mutations detected in any of the specimens.

**Conclusions:**

These results indicate that elevated expressions of the tumor-related genes FPGS/GGH and VEGF are correlated with malignancy of TC and B3 tumors. Additional examinations will be necessary to investigate their chemosensitivity.

## Background

A thymic carcinoma (TC) is a rare type of malignant neoplasm that develops in the anterior mediastinum and associated with poor prognosis [1]. Although all types of thymoma have a malignant potential, type B3 (B3), especially in an advanced stage, shows a poor prognosis, the same as TC [1, 2]. Complete surgical resection is the preferred treatment to achieve a cure in B3 cases, while patients with metastatic or recurrent tumors are candidates for systemic chemotherapy. Platinum-based protocols are used for first-line chemotherapy to treat these thymic epithelial tumors, and recently the expressions of various tumor-related genes have been reported to correlate with the chemo-sensitivity of several types of cancer and prognosis of affected patients [3–6]. Furthermore, several studies have compared gene expression levels in TC and thymoma cases [7]. In the present study, we investigated the expressions of tumor-related genes in these rare tumors to establish an appropriate treatment strategy.

## Methods

Specimens resected from consecutive patients with TC or B3 who underwent surgery from January 1999 through April 2012 at our institutions were investigated. To compare with lung cancer (LC) tumors, several characteristics of LC cases in our database [8, 9] were also examined. We found 49 cases of adenocarcinoma (AD) and 49 of squamous cell carcinomas (SCC) in the database, and used them for comparisons. Of the ADs, 26, 8, and 15 were stage I, II, and III, respectively, while those stages were seen in 32, 7, and 10, respectively, of the SCC cases. Sixteen patients with AD and 12 with SCC had a relapse during the observation period. The Dokkyo Medical University Hospital Ethics Committee approved this retrospective study (#24036) and waived the need for patient consent for analysis of the results.

### Determination of tumor-related gene expression

Excised specimens were thinly sliced, then tumor segments were collected by micro-dissection to extract mRNA. Real time polymerase chain reaction (RT-PCR) assays were performed according to Danenberg’s tumor profile (DTP) method [10] for semi-quantitation of mRNA of tumor-related genes (Response Genetics Inc., New York, USA), including thymidylate synthase (TS), dihydropyrimidine dehydrogenase (DPD), thymidine phosphorylase (TP), dihydrofolate reductase (DHFR), gamma-glutamyl hydrolase (GGH), folypolyglutamyl synthase (FPGS), vascular endothelial growth factor (VEGF), excision repair cross-complementation group 1 (ERCC1), topoisomerase-1 (TOPO1), and epidermal growth factor receptor (EGFR). In brief [8, 10], a laser-captured micro-dissection technique was used with 10-μm sections at a magnification of 100x to obtain only cancer cells. Next, 1500–2000 round spots 80 μm in diameter were dissected for each case, from which total RNA was extracted. Real-time quantitative reverse transcription-PCR assays were performed with those samples using an ABI 7700 and TaqMan Probes. The level of ß-actin mRNA was used as a reference. Relative gene expression values are expressed as the ratio of PCR products of the gene of interest to that of ß-actin, the internal reference gene.

### EGFR mutation

Tumor tissues were obtained from the patients and placed as formalin-fixed paraffin-embed samples on slide glasses. Detection of the EGFR mutation in each specimen was performed using a peptide nucleic acid-locked nucleic acid (PNA-LNA) PCR clamp assay (SRL Inc., Tokyo, Japan).

### Statistics

Values are shown as the mean ± SD. An unpaired *t* test with Welch correction was used for comparing between 2 groups. For non-parametric data, a Mann–Whitney test was used. Analysis of variance (ANOVA) was utilized for comparing among 3 or more groups, then Tukey’s post-hoc test was used when significance was found. Survival rate was calculated by the Kaplan-Meier method. A log-rank test was used for comparisons among groups. Receiver operating characteristic (ROC) curve analysis was done to estimate optimal sensitivity and specificity for prediction of overall survival according to different cutoff levels of relative gene expression value. Statistical calculations were performed using GraphPad Prism 6 (GraphPad Software Inc., La Jolla, CA. USA). Differences were considered significant at *P* < 0.05.

## Results

Among 76 patients who underwent surgery for thymic epithelial tumors at our institution, 14 had TC (18.4 %) and 8 had B3 (10.5 %). Patient characteristics are shown in Table 1.

**Table 1:** Patient characteristics in thymic cancer and B3 thymoma groups

	Thymic cancer	B3 thymoma
Sex		
Male/female	7/7	3/5
Age in years (range)	61.5 (36–82)	51.5 (36–78)
Induction therapy	0/14	0/8
Status at time of study		
Alive	2	6
Alive with relapse	2	1
Dead	10	1

### Nucleotide-metabolism-related enzymes

**Fig. 1:**
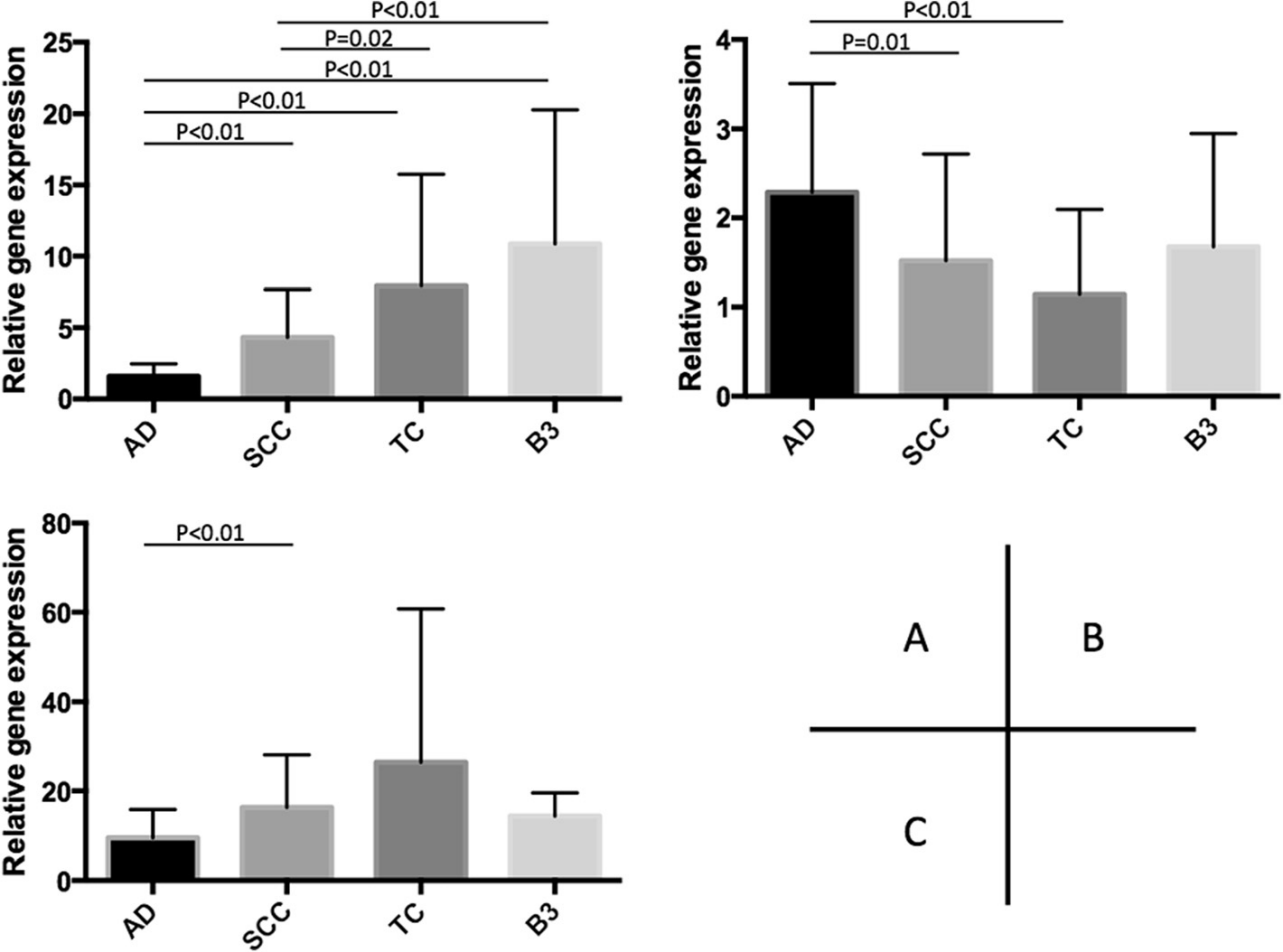
Comparison of relative gene expression of nucleotide-metabolism-related enzymes. **a** thymidylate synthase, **b** dihydropyrimidine dehydrogenase, **c** thymidine phosphorylase. AD, lung adenocarcinoma (*n* = 49); SCC, lung squamous cell carcinoma (*n* = 49); TC, thymic cancer (*n* = 14); B3, B3 thymoma (*n* = 8)

Gene expression levels of TS, DPD, and TP in the TC specimens were compared to those in the B3 specimens, and also compared to those in lung ADs and SCCs. The relative gene expression value of TS was 7.93 ± 7.82 in TC, 10.87 ± 9.41 in B3, 1.60 ± 0.86 in lung AD, and 4.33 ± 3.35 in lung SCC (Fig. 1a). That value was significantly lower in lung AD as compared to the others (*p* < 0.001), and lower in lung SCC as compared to both TC (*p* = 0.02) and B3 (*p* < 0.01). The relative gene expression value of DPD was 1.14 ± 0.95 in TC, 1.67 ± 1.28 in B3, 2.29 ± 1.22 in lung AD, and 1.52 ± 1.20 in lung SCC (Fig. 1b). That value was significantly higher in lung AD as compared to both lung SCC (*p* = 0.01) and TC (*p* < 0.01). The relative gene expression value of TP was 26.42 ± 34.36 in TC, 14.36 ± 5.18 in B3, 9.52 ± 6.30 in lung AD, and 16.27 ± 11.84 in lung SCC (Fig. 1c). That value was significantly lower in lung AD as compared to lung SCC (*p* < 0.01).

### Folate-metabolism-related enzymes

**Fig. 2:**
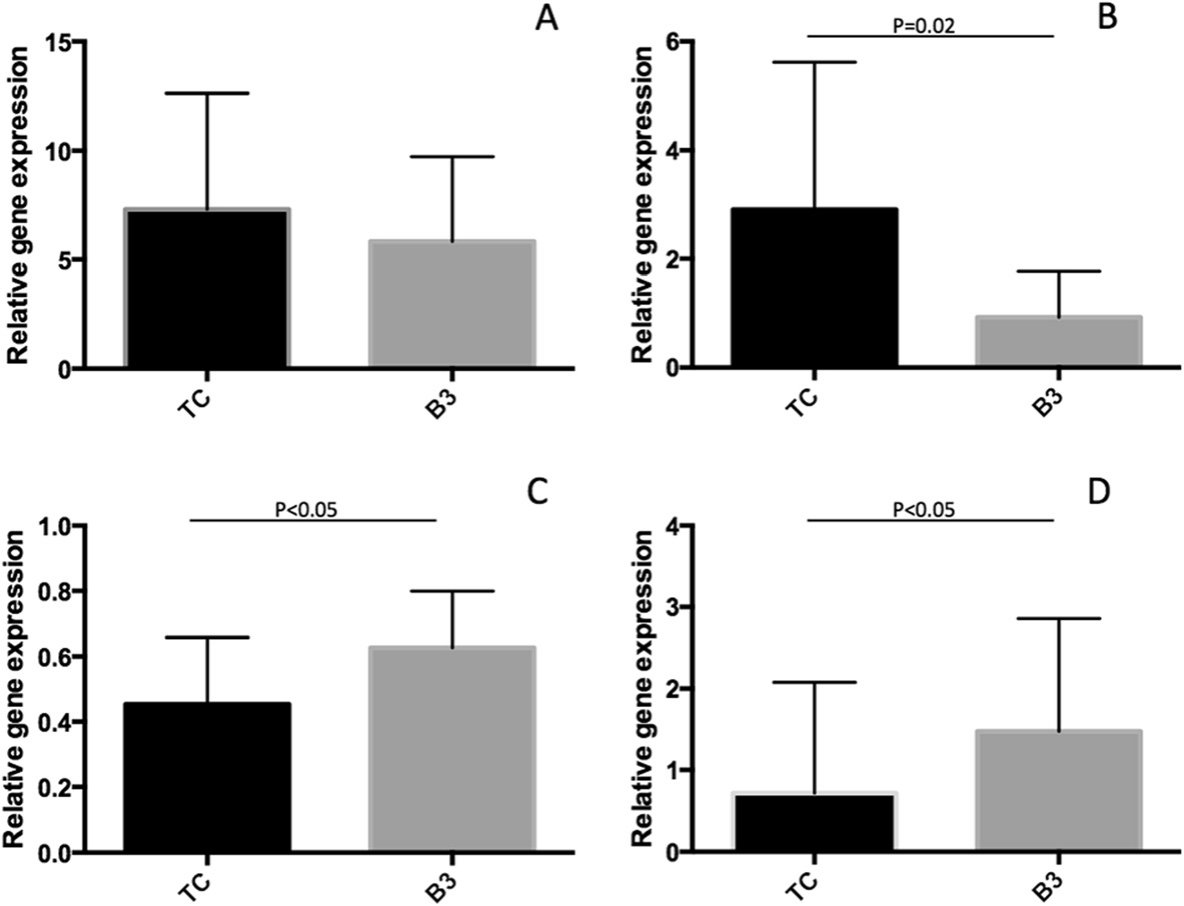
Comparison of relative gene expression of folate-metabolism-related enzymes. **a** dihydrofolate reductase (DHFR), **b** gamma-glutamyl hydrolase (GGH), **c**: folypolyglutamyl synthase (FPGS), **d** FPGS/GGH. TC, thymic cancer (*n* = 14); B3, B3 thymoma (*n* = 8 for DHFR, *n* = 7 for others)

Gene expression levels of DHFR, GGH, and FPGS in the TC specimens were compared to those in the B3 specimens. The relative gene expression value of DHFR was 7.31 ± 5.33 in TC and 5.83 ± 3.90 in B3 (Fig. 2a), which was not significantly different. The relative gene expression value of GGH was 2.91 ± 2.71 in TC and 0.93 ± 0.84 in B3 (Fig. 2b), which was significantly higher in the TC specimens (*p* = 0.02). The relative gene expression value for FPGS was 0.45 ± 0.20 in TC and 0.63 ± 0.17 in B3 (Fig. 2c), which was significantly different (*p* < 0.05). The ratio of FPGS and GGH (FPGS/GGH) was 0.72 ± 1.36 in TC and 1.48 ± 1.39 in B3 (Fig. 2d), significantly lower in the TC specimens (*p* < 0.05).

### Other tumor-related gene expressions

**Fig. 3:**
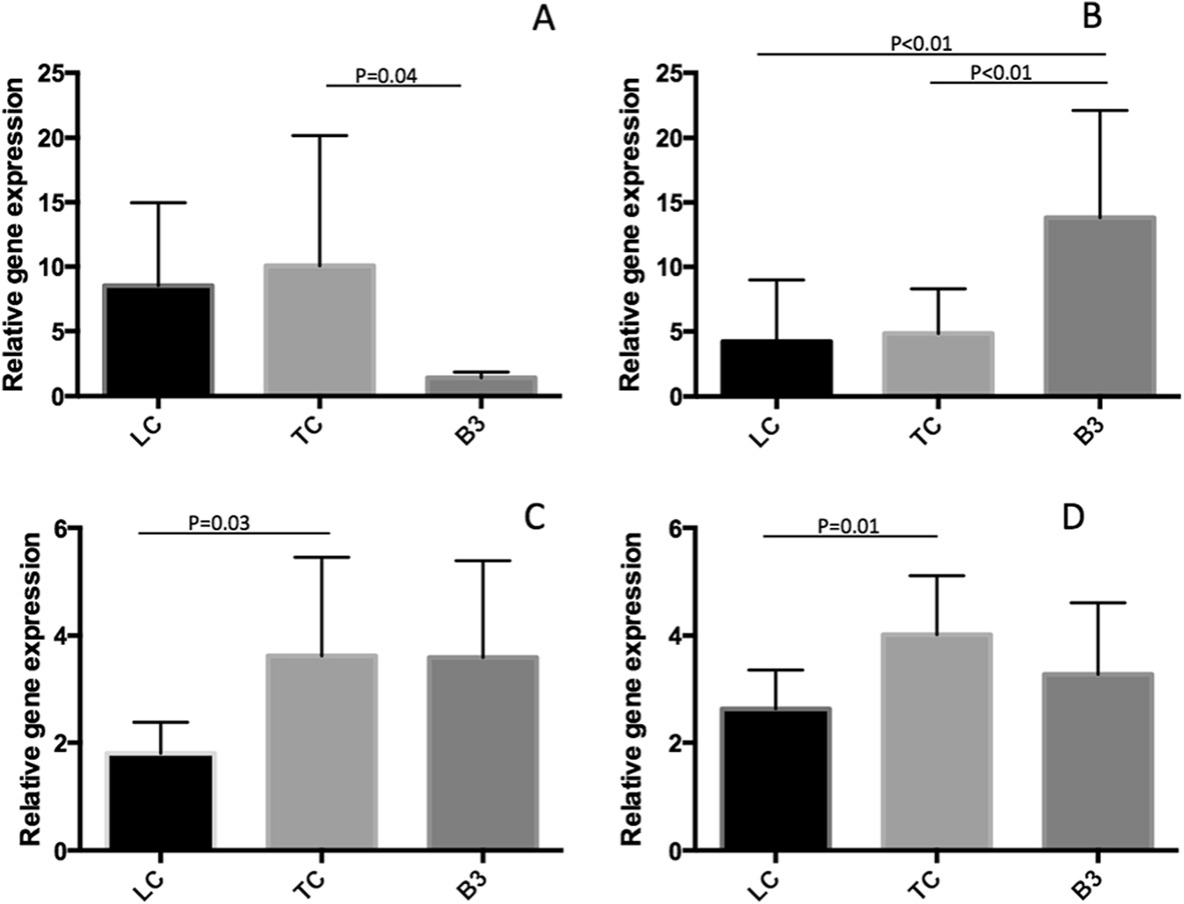
Expressions of other genes. **a** vascular endothelial growth factor, **b**: epidermal growth factor receptor, **c** excision repair cross-complementation group 1, **d** topoisomerase-1. LC, lung cancer (*n* = 9); TC, thymic cancer (*n* = 14); B3, B3 thymoma (*n* = 8)

The relative gene expression values for VEGF, ERCC1, TOPO1, and EGFR in the TC and B3 specimens were compared to those in LC specimens. The relative gene expression value for VEGF was 10.1 ± 10.1 in TC, 1.42 ± 0.46 in B3, and 7.72 ± 6.26 in LC (Fig. 3a), which was significantly lower in B3 as compared to TC (*p* = 0.04). The relative gene expression value for EGFR was 4.85 ± 3.45 in TC, 13.8 ± 8.3 in B3, and 4.22 ± 4.77 in LC (Fig. 3b), which was significantly higher in B3 as compared to TC (*p* < 0.01) and LC (*p* < 0.01). On the other hand, there were no EGFR gene mutations found in the TC and B3 specimens. The relative gene expression value for ERCC1 was 3.62 ± 1.83 in TC, 3.60 ± 1.79 in B3, and 1.80 ± 0.58 in LC (Fig. 3c), which was significantly higher in TC as compared to LC (*p* = 0.03). The relative gene expression value for TOPO1 was 4.02 ± 1.10 in TC, 3.28 ± 1.34 in B3, and 2.63 ± 0.73 in LC (Fig. 3d), which was significantly higher in TC as compared to LC (*p* = 0.01).

### Analysis of survival in relationship to gene expressions

**Fig. 4:**
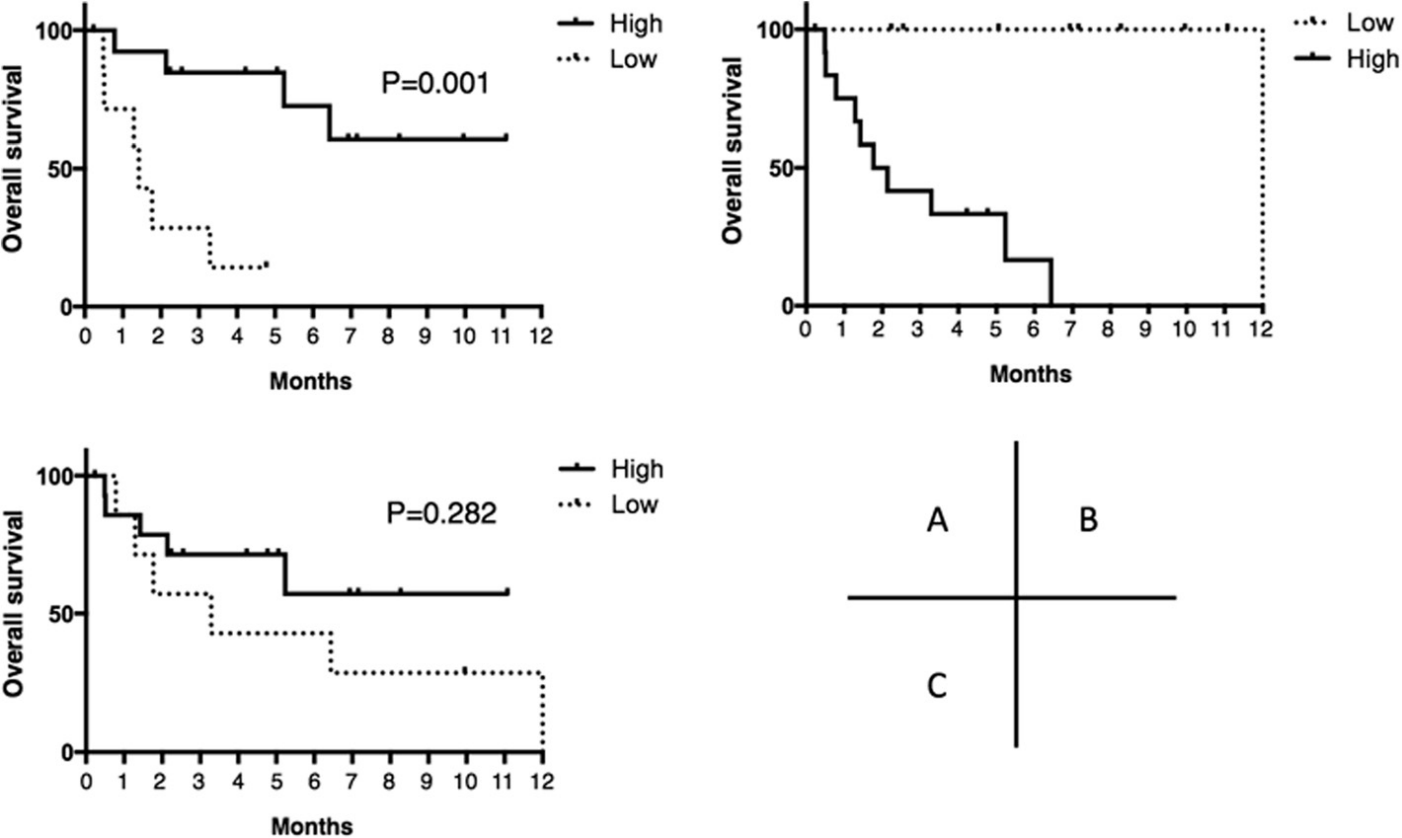
Overall survival in relation to gene expression. **a** ratio of folypolyglutamyl synthase to gamma-glutamyl hydrolase (FPGS/GGH), High, ≥0.21 (*n* = 14); Low, <0.21 (*n* = 7), **b** vascular endothelial growth factor (VEGF), High, >2.19 (*n* = 12); Low, ≤2.19 (*n* = 10), **c** excision repair cross-complementation group 1 (ERCC1), High, ≥2.52 (*n* = 15); Low, <2.52 (*n* = 7)

As described above, the gene expression levels of FPGS, GGH, VEGF, and EGFR were different between TC and B3. To determine whether relative gene expression has an effect on overall survival, the cut-off values for FPGS/GGH, VEGF, and EGFR were calculated using ROC curve analysis and shown to be 0.21, 2.19, and 2.52, respectively. Overall survival curves are presented in Fig. 4. A higher value for FPGS/GGH was related to significantly superior survival as compared to a lower value (*p* = 0.001), while a lower value for VEGF was related to significantly superior survival as compared to a higher value (*p* = 0.0001). There were no statistically significant differences regarding survival between higher and lower values of EGFR (*p* = 0.282).

## Discussion

Among thymic epithelial tumors, TC is recognized as a distinct entity from thymoma [1], while B3 is considered to be a low-grade malignant tumor, and intermediate between TC and other types of thymoma [1, 2]. Patients with metastatic or recurrent TC or B3 tumors are candidates for systemic chemotherapy, and platinum-based protocols are standard first-line chemotherapy options for thymic tumors [4, 11]. In the present study, we investigated tumor-related gene expressions of TC and B3. As for nucleotide-metabolism-related enzymes, TC and B3 had a higher gene expression level of TS as compared to lung AD and SCC. As for folate-metabolism-related enzymes, the FPGS/GGH ratio was higher in B3 as compared to TC, while VEGF gene expression was much higher in TC than in B3. Furthermore, a higher value for FPGS/GGH gene expression and a lower value for VEGF were associated with superior overall survival. EGFR gene expression was higher in B3 than in TC, though no EGFR mutations were found in either the B3 or TC specimens.

Gene expression levels of the nucleotide-metabolism-related enzymes TS, DPD, and TP in TC and B3 were compared to those in lung AD and SCC cases listed in our database. Anti-metabolic drugs, such as pemetrexed, gemcitabine, and 5-fluorouracil and its derivatives, are widely used as cancer chemotherapy agents, and their effects include inhibition of TS, as well as incorporation of its metabolites into RNA and DNA. TP catalyzes the synthesis of active metabolites, resulting in disruption of the activities of TS and DNA, as well as RNA synthesis. Lower TS activity is thought to be correlated with greater sensitivity to anti-metabolic drugs [8, 10]. The present findings showed that gene expression levels of TS mRNA in TC and B3 were significantly higher than in lung AD and SCC. Furthermore, the level of DPD mRNA in TC was lower than in lung AD, while it was similar to that in lung SCC. The gene expression level of TP mRNA in TC and B3 was not different as compared to that in LC, whereas that level in lung AD was lower than in lung SCC. Generally, lung AD is thought to be more sensitive to anti-metabolic drugs as compared to lung SCC. Although our results do not directly show the level of chemosensitivity of these tumors, they indicate that TC and B3 are likely not as sensitive to anti-metabolic drugs as lung AD.

We also compared gene expression levels of the folate-metabolism-related enzymes DHFR, GGH, and FPGS, as well as the FPGS/GGH ratio between TC and B3. Folate deficiency increases the risk of cancer development by induction of an imbalance in DNA precursors and altering DNA methylation [12]. DHFR is a target enzyme of methotrexate (MTX), an inhibitor of folate metabolism. Kawakami et al. reported that the mRNA level of DHFR in a tumor was correlated with chemo-sensitivity for MTX [13]. FPGS converts reduced-folate monoglutamate to polyglutamate in an intracellular manner, which results in retention of MTX-polyglutamate inside the cells. On the other hand, GGH catalyzes the degradation of inter- and intracellular polyglutamate [14]. Folate polyglutamate regulates the reaction rate of the key enzyme for metabolism, thus FPGS/GGH ratio is important for folate metabolism, with a higher ratio thought to be correlated with greater sensitivity to anti-metabolic drugs including anti-folate agents [14]. We found that a higher FPGS/GGH gene expression ratio value was associated with significantly superior survival as compared to a lower value, with a cutoff value of 0.21. Therefore, it is likely that FPGS/GGH ratio plays an important role in survival of patients with TC and B3, and that B3 is more sensitive to anti-folate drugs as compared to TC.

VEGF is a signal protein that stimulates angiogenesis and anti-VEGF therapies are important for treatment of certain cancers, because solid tumors do not grow beyond a limited size without an adequate blood supply provided by angiogenesis using VEGF signals. VEGF gene expression in TC was higher than that in B3, while higher VEGF gene expression in TC and B3 was related to worse overall survival. These results indicate that VEGF mRNA expression is correlated with malignancy, thus anti-VEGF therapy may be a potential candidate for patients with higher levels of VEGF gene expression.

Several studies have reported that EGFR is overexpressed in thymomas and TCs, with higher EGFR staining significantly associated with stage III-IV cases [3, 15]. The level of EGFR mRNA was higher in B3 than in both TC and LC in the present study. However, no EGFR mutations were detected in the B3 and TC specimens. Several previous reports have also noted that EGFR mutations are rare in thymic malignancies [3, 16]. Furthermore, Nakagiri et al. reported a case in which gefitinib therapy failed to reduce tumor size and also noted an EGFR mutation caused by exon 19 deletion [17]. Thus, an EGFR-tyrosine kinase inhibitor is an unlikely candidate for treatment of TC and B3 patients.

ERCC1 protein is involved in nucleotide excision repair of damaged DNA and determination of ERCC1 mRNA expression may be clinically useful for cancer treatment, as one of the mechanisms of resistance to platinum chemotherapy drugs has been shown to be correlated with high ERCC1 activity [18, 19]. We found that the gene expression level of ERCC1 mRNA was higher in the present TC specimens as compared to LC. However, our findings are limited and do not fully reveal the efficacy of platinum-based chemotherapy, though cisplatin is considered to be a candidate for TC treatment. Furthermore, TOPO-1, which is involved in cell division, is a target of some anti-cancer drugs, including irinotecan, topotecan, and camptothecin. Several reports have shown that higher TOPO-1 protein levels are correlated with good clinical outcome [20]. In the present TC specimens, TOPO-1 gene expression was higher as compared to the LC specimens.

The present study is limited by its design as an institutional report and the small population, due to rarity of the disease. Additional studies and case accumulation are necessary.

## Conclusion

Tumor-related gene expressions were analyzed in TC, B3, and LC specimens. Folate metabolism, as represented by FPGS/GGH gene expression ratio, was different between TC and B3, and a higher ratio was associated with significantly superior survival as compared to a lower ratio. Furthermore, VEGF gene expression in TC was higher than that in B3, while higher VEGF gene expression in TC and B3 was related to worse overall survival. These results indicate that these gene expressions are correlated with malignancy in TC and B3 cases. Additional examinations are necessary to evaluate the chemosensitivity of these tumors.
